# 339. COVID-19 Mortality in a Private Hospital in Mexico City

**DOI:** 10.1093/ofid/ofab466.540

**Published:** 2021-12-04

**Authors:** Maria Lorena Cabrera Ruiz, Paulo Castañeda-Méndez, Daniel Aguilar-Zapata, Javier Reyes Mar, Gonzalez Chon Octavio, Luis E Soto-Ramirez

**Affiliations:** 1 Hospital Medica Sur, Ciudad de México, Distrito Federal, Mexico; 2 Hospital Medica Sur / Hospital San Angel Inn Universidad, Mexico city, Distrito Federal, Mexico; 3 MEDICA SUR, Mexico City, Distrito Federal, Mexico

## Abstract

**Background:**

According to the Institute of Global Health Science (IGHS), mortality for Covid-19 patients treated in public hospitals in Mexico ranges between 30-50%, decreasing to 20% in private health care facilities. Our objective was to describe the mortality rate in a teaching private hospital in Mexico City.

**Methods:**

We included all patients that were admitted to hospital Medica Sur, in the south part of Mexico City during year 2020. We analyzed the total mortality presented in all our patients with a follow up of two months, and relay that to age and gender.

**Results:**

During year 2020, we admitted in our hospital 1,075 patients with confirmed diagnosis of COVID-19 through nasopharyngeal molecular test; 772 were male (71.8%) with more than 50% between 40 and 59 years, while females were more frequent between 40 and 69 years’ age. Seventy-four patients (6.88%) died during hospitalization; 59 (79.7%) males and 15 females. Mortality rate was clearly related to age (figure 1) with 30% mortality for males between 80-89 years and 19% for females.

Mortality rate by gender and age

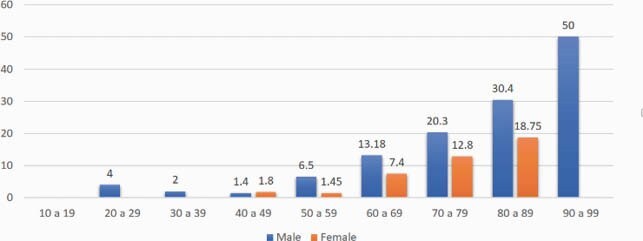

**Conclusion:**

Mortality in private hospitals was clearly lower than in public hospitals. In our hospital, mortality was lower than 10%, mostly related to their availability of unlimited intensive care without ECMO and despite the lack of some drugs like Remdesivir. As described, space limitations for intensive care as well as the lack of trained personal impacted significantly the mortality in public hospitals.

**Disclosures:**

**All Authors**: No reported disclosures

